# Modulation of flavonoid biosynthetic pathway genes and anthocyanins due to virus infection in grapevine (*Vitis vinifera *L.) leaves

**DOI:** 10.1186/1471-2229-10-187

**Published:** 2010-08-23

**Authors:** Linga R Gutha, Luis F Casassa, James F Harbertson, Rayapati A Naidu

**Affiliations:** 1 Department of Plant Pathology, Irrigated Agriculture Research and Extension Center, Washington State University, Prosser, WA 99350, USA; 2 School of Food Science, Irrigated Agriculture Research and Extension Center, Washington State University, Prosser, WA 99350, USA

## Abstract

**Background:**

Symptoms of grapevine leafroll disease (GLRD) in red-fruited wine grape (*Vitis vinifera *L.) cultivars consist of green veins and red and reddish-purple discoloration of inter-veinal areas of leaves. The reddish-purple color of symptomatic leaves may be due to the accumulation of anthocyanins and could reflect an up-regulation of genes involved in their biosynthesis.

**Results:**

We examined six putative constitutively expressed genes, *Ubiquitin, Actin*, *GAPDH*, *EF1-a, SAND *and *NAD5*, for their potential as references for normalization of gene expression in reverse transcription-quantitative real-time polymerase chain reaction (RT-qPCR). Using the *geNorm *program, a combination of two genes (*Actin *and *NAD5*) was identified as the stable set of reference genes for normalization of gene expression data obtained from grapevine leaves. By using gene-specific RT-qPCR in combination with a reliable normalization factor, we compared relative expression of the flavonoid biosynthetic pathway genes between leaves infected with *Grapevine leafroll-associated virus 3 *(GLRaV-3) and exhibiting GLRD symptoms and virus-free green leaves obtained from a red-fruited wine grape cultivar (cv. Merlot). The expression levels of these different genes ranged from two- to fifty-fold increase in virus-infected leaves. Among them, *CHS3*, *F3'5'H*, *F3H1*, *LDOX*, *LAR1 *and *MybA1 *showed greater than 10-fold increase suggesting that they were expressed at significantly higher levels in virus-infected symptomatic leaves. HPLC profiling of anthocyanins extracted from leaves indicated the presence of cyanidin-3-glucoside and malvidin-3-glucoside only in virus-infected symptomatic leaves. The results also showed 24% higher levels of flavonols in virus-infected symptomatic leaves than in virus-free green leaves, with quercetin followed by myricetin being the predominant compounds. Proanthocyanidins, estimated as total tannins by protein precipitation method, were 36% higher in virus-infected symptomatic leaves when compared to virus-free green leaves.

**Conclusions:**

The results, the first example to our knowledge, showed that modulation of the flavonoid biosynthetic pathway occurred in GLRaV-3-infected leaves of a red-fruited wine grape cultivar (cv. Merlot) leading to *de novo *synthesis of two classes of anthocyanins. These anthocyanins have contributed to the expression of reddish-purple color of virus-infected grapevine leaves exhibiting GLRD symptoms.

## Background

**Figure 1:**
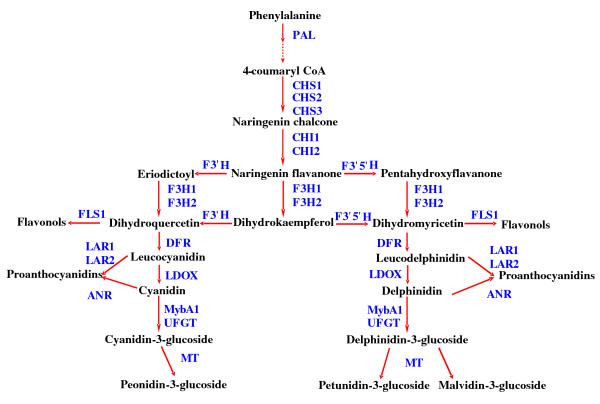
**Schematic representation of the flavonoid biosynthetic pathway**. The pathway is drawn based on information from Hummer and Schreier and Boss *et al*. [59,81]. *PAL*, phenylalanine ammonia-lyase; *CHS1, CHS2*, and *CHS3*, chalcone synthase 1, 2, and 3, respectively; *CHI1 *and *CHI2*, chalcone isomerase 1 and 2, respectively; *F3'H *-flavonoid-3'-hydroxylase; *F3'5'H *- flavonoid-3', 5'-hydroxylase; *F3H1 *and *F3H2*, flavanone-3-hydroxylase 1 and 2, respectively; *DFR*- dihydroflavonol reductase; *LDOX*- leucoanthocyanidin dioxygenase; *UFGT*, UDP-glucose:flavonoid 3-*O*-glucosyltransferase, *FLS1*, flavonol synthase 1, *LAR1 *and *LAR2*, leucoanthocyanidin reductase 1 and 2, respectively; *ANR*, anthocyanidin reductase; *MT*, methyl transferase; *MybA1*, MYB transcription factor gene.

In plants, three major classes of flavonoids (anthocyanins, proanthocyanidins and flavonols) are synthesized via the branched flavonoid biosynthetic pathway [1,2]. These secondary metabolites contribute to the 'colorful' pigmentation of flowers, fruits, seeds and leaves and are involved in several physiological and biochemical processes in plants such as UV protection, insect attraction, herbivore defense and symbiosis [3-5]. Plants also utilize various colors conferred by anthocyanins to recruit pollinators and attract animals to disperse seeds [2]. The flavonoids are often produced in vegetative tissues as well under stress conditions, such as high light intensity, cold temperature, nutrient deficiency and pathogen attack or senescence [6-8]. Due to a multitude of biological and agricultural importance and favorable health benefits, the genetics and biochemistry of the flavonoid biosynthetic pathway has been intensively studied in several plant species [9-11]. These studies indicated that flavonoid composition among plant species and even different tissues of a plant can be remarkably different [1,12-15]. Further details about the flavonoid biosynthetic pathway are available in many publications [1-3,5]. A generalized scheme of the pathway is shown in Figure 1.

For many years, berries of the grapevine (*Vitis vinifera *L.) have received more attention due to their significance as an important edible source of flavonoid compounds with nutrient and health benefits for humans [16]. Different flavonoid compounds are largely localized in berry skin and play a critical role in the quality of wine by contributing to its astringency and color [2,3]. The major flavonoid classes accumulated in red-fruited grapevine berries are flavonols, proanthocyanidins (also called condensed tannins) and anthocyanins, with anthocyanins accumulating mostly in berry skin and the tannins in seed [17,18]. Thus, the flavonoid biosynthetic pathway in berries is regulated in a temporal and tissue-specific manner and the expression pattern of the pathway genes correlates to the synthesis of flavonoids in different grapevine berry tissues during fruit development [14,19]. The synthesis of flavonoids via the flavonoid biosynthetic pathway requires two classes of genes: structural genes that encode enzymes for synthesis of anthocyanins and other flavonoids, and the regulatory genes involved in spatial and temporal regulation of these structural genes [20]. Although these two classes of genes are present in both red- and white-fruited grapevine cultivars, the color pigments are not expressed in white-fruited cultivars due to multiallelic mutations in the regulatory genes called *MybA1 *and *MybA2 *[21-24]. These two genes regulate expression of the UDP-glucose:flavonoid 3-*O*-glucosyltransferase (*UFGT*) gene, which mediates the conversion of anthocyanidins to anthocyanins by glycosylation [25,26]. Thus, the last biosynthetic step of *UFGT*-mediated anthocyanin synthesis does not occur in white-fruited grapevine cultivars and hence these cultivars do not express color in their berries. In the case of red-fruited berries, anthocyanins are transported into vacuoles and ultimately accumulated in berry skin cells [25,27]. In general, berries from red-fruited cultivars show various grades of color depending on the quantity and composition of anthocyanins in the berry skin. Proanthocyanidins are synthesized mainly at the green stage of berry development, whereas synthesis of anthocyanins begins at *véraison *(a transitional phase of grapevine berry development representing the beginning of berry ripening) and continue to accumulate in berry skins during ripening [17,27,28]. Although anthocyanins are present largely in berry skins of red-fruited grapevine cultivars, they can also accumulate in some cases in various plant organs such as leaves, flowers, stems, tendrils and berry flesh [29].

Grapevine leafroll disease (GLRD) is the most serious and complex virus disease known to infect grapevines worldwide [30]. Up to ten serologically distinct viruses, termed grapevine leafroll-associated viruses (GLRaVs) and numbered sequentially GLRaV-1 to -10 in the order of their discovery, have thus far been documented in grapevines infected with GLRD [31]. GLRaVs are flexuous rods, 1400-2200 nm long and 10-12 nm diameter with a monopartite, positive sense, single-stranded RNA genome. They are phloem-limited and predominantly dispersed long distances via clonally propagated vegetative planting materials. Some of the currently documented GLRaVs have been shown to be spread by different species of mealybugs and scale insects [30]. Among them, GLRaV-3 (genus *Ampelovirus*, family *Closteroviridae*) is the most economically important and widely prevalent. The virus has the largest genome size (18,498 nucleotides) encoding 13 open reading frames and represents the most complex gene organization among the currently known closteroviruses infecting grapevines [32].

It has been documented in several grape-growing regions that GLRaV-3 can cause reduced plant vigor and longevity, and significant losses in both yield and quality of berries [33-35]. In red-fruited wine grape cultivars infected with GLRaV-3, mature leaves at the bottom portions of canes begin to show GLRD symptoms at or soon after *véraison*. As the season progresses, the symptoms extend upward to other leaves and the foliar discolorations expand and coalesce to form a reddish-purple color within the inter-veinal areas of the leaf; a narrow strip of leaf tissue often remains green on either side of the main veins (hence called green veins). By the later part of the season (August-October), a typical infection in a red-fruited cultivar will consist of green veins and red and reddish-purple coloration of inter-veinal areas [30]. In advanced stages, the margins of infected leaves roll downward, expressing the symptom that gives the disease its common name. White-fruited cultivars may express GLRD symptoms as mild yellowing or chlorotic mottling and, in some cases, leaf margins may roll downward toward the end of the season. Unlike white-fruited cultivars, the phenotypic expression of reddish-purple coloration of leaves in red-fruited cultivars due to GLRD may be an indication of the accumulation of anthocyanins and could reflect the up-regulation of genes involved in their biosynthesis in GLRaV-3-infected symptomatic leaves. However, no studies have been conducted to elucidate the expression pattern of flavonoid biosynthetic pathway genes or analyze different flavonoids in grapevine leaves showing GLRD symptoms.

In recent years, reverse transcription-quantitative real-time polymerase chain reaction (RT-qPCR) has been widely employed as a powerful tool for investigating the expression of cellular genes in response to biotic and abiotic stresses [36,37]. Throughout the manuscript, we used the abbreviation qPCR for quantitative real-time polymerase chain reaction, RT-qPCR for reverse transcription-qPCR and RT-PCR for 'traditional' RT-PCR. Due to its high throughput nature, sensitivity and accuracy in quantifying target genes, RT-qPCR is capable of the relative or absolute quantification of target genes in a given sample over a large dynamic range of conditions [38]. Considering its ability to discriminate between the expression of closely related genes and to quantify very weekly expressed genes, RT-qPCR is considered particularly useful for elucidating molecular mechanisms that underlie changes in gene expression [39,40]. Even though RT-qPCR is a method of choice, the reliability and reproducibility of experimental results for quantitative gene expression is dependent on the quality of RNA template and cDNA, primer specificity, assay efficiency, experimental conditions and rigorous analysis of the data using appropriate quality controls [41,42]. To circumvent bias, normalization of relative quantities of the target genes is carried out widely using appropriate endogenous reference genes, also referred in earlier studies as housekeeping genes [43,44]. One of the most critical issues in RT-qPCR is the choice of reference genes used for gene expression analysis, since the expression of a number of such reference genes varies considerably under each experimental condition in different lab settings [45-48]. Validation of reference genes is necessary, since the use of a single non-validated reference gene has been shown to significantly increase bias in experimental validation of gene expression changes ranging from more than 3-fold in 25% of the results up to 6-fold in 10% of the results [43].

In this study, we evaluated six reference genes for their use in gene expression studies in virus-free green leaves and virus-infected leaves exhibiting GLRD symptoms. Using RT-qPCR assay, based on SYBR green detection, we analyzed expression stability of these reference genes in grapevine leaves using the *geNorm *algorithm [43]. A combination of two genes was identified as suitable candidates for normalization of gene expression data in both virus-free and virus-infected leaves. By using gene-specific RT-qPCR, in combination with a reliable normalization factor, we present evidence that up-regulation of flavonoid biosynthetic pathway genes occurred in symptomatic leaves of a red-fruited wine grape cultivar infected with GLRaV-3. Together with estimation of anthocyanins, flavonols and proanthocyanidins, these results indicated modulation of the flavonoid biosynthetic pathway genes towards accumulation of certain classes of end-products in grapevine leaves exhibiting GLRD symptoms.

## Results

### GLRD symptoms in grapevine leaves

**Figure 2:**
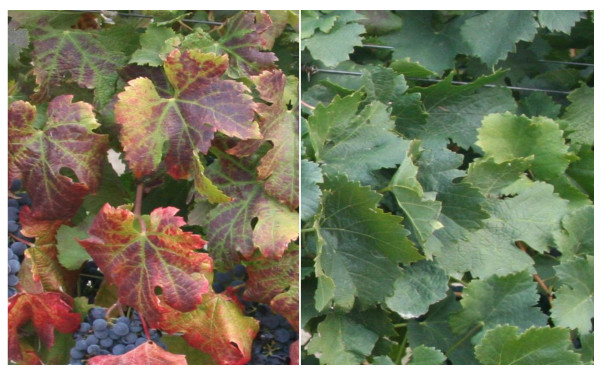
**GLRD symptoms in GLRaV-3-infected red-fruited wine grape cv. Merlot**. Picture on the left shows leaves from GLRaV-3-infected grapevine showing green veins and red and reddish-purple discoloration between inter-veinal areas and downward rolling of leaf margins and picture on the right shows green leaves from an adjacent virus-free grapevine.

At the time of sampling in mid September, representing post-*véraison *stage of berry development, mature leaves at the bottom portion of canes in GLRD affected Merlot grapevines showed green veins and red and reddish-purple color in the inter-veinal areas (Figure 2, left). The margins of some of these leaves showed downward rolling. GLRD symptoms were not observed in adjacent grapevines (Figure 2, right). Symptomatic leaves from GLRD affected grapevines and comparable leaves from adjacent grapevines not affected by GLRD were tested by single tube-one step RT-PCR for the presence of different grapevine viruses. Symptomatic leaves from GLRD affected grapevines were tested positive for GLRaV-3 but not for other viruses (data not shown). GLRaV-3 was detected in green veins as well as in reddish-purple inter-veinal areas (since minor veins and veinlets are present in these areas) of symptomatic leaves from GLRD affected grapevines (Additional file 1, Figure S1). Green leaves from adjacent grapevines were tested negative for these viruses.

### Chlorophyll and carotenoid pigments in symptomatic leaves

Total chlorophylls and carotenoids were estimated in GLRaV-3-infected symptomatic and virus-free green leaves (Table 1). Total chlorophyll content in symptomatic leaves was less by 20.1% when compared to green leaves. Similarly, total carotenoids were less by 19.8% in virus-infected symptomatic leaves. These results indicate reduced levels of both chrolophylls and carotenoids in virus-infected leaves exhibiting GLRD symptoms.

**Table 1:** Total chlorophylls and carotenoids in GLRaV-3-infected symptomatic and virus-free green leaves

Pigments (mg/g fresh wt)	GLRaV-3-infected*	Virus-free*
**Total chlorophylls**	4.81 ± 0.45	6.02 ± 0.16
**Total carotenoids**	2.19 ± 0.19	2.73 ± 0.08

*Values are mean ± SE. Asterisk indicates significant difference between GLRaV-3-infected and virus-free leaves using one way ANOVA test (*P < 0.05)

### Sequence specificity and amplification efficiency analysis of target genes

In initial experiments using total RNA isolated from grapevine leaves, gene-specific sequences amplified by RT-PCR were cloned and nucleotide sequence determined (Table 2). Nucleotide sequence obtained for each gene showed high level of similarity (97 to 100%) with corresponding gene sequence available in GenBank, confirming the specificity of each amplicon to the respective gene. The amplification efficiency (E) of each gene-specific primer pair in RT-qPCR shown in Table 2 indicated the suitability of primer pairs for RT-qPCR-based amplification and quantification of target genes. Melting curve analysis for each amplicon showed a single peak (Additional file 2, Figure S2), further confirming the homogeneity and specificity of amplicons produced in qPCR for all target genes. Agarose gel electrophoretic separation of each amplicon showed a single DNA fragment of the expected size with no visible primer-dimer products (data not shown). No amplifications were observed in all control assays. All these results indicated that the total RNA and the derived cDNA template were free of contaminating genomic DNA, demonstrating high quality of nucleic acid preparations obtained for gene expression level analyses by RT-qPCR.

**Table 2:** Genes, primers, length of amplicons and amplification efficiency

Gene^1^	Primer sequence 5'-3' (forward/reverse)	Amplicon length (bp)	qPCR efficiency	Reference	GenBank accession number*
**a) Reference genes**					
*Ubiquitin*	TCTGAGGCTTCGTGGTGGTA/AGGCGTGCATAACATTTGCG	99	2.16	[82]	GU585868
*Actin*	CTTGCATCCCTCAGCACCTT/TCCTGTGGACAATGGATGGA	82	2.11	[56]	GU585869
*GAPDH*	TTCTCGTTGAGGGCTATTCCA/CCACAGACTTCATCGGTGACA	70	1.84	[56]	GU585870
*EF1-a*	GAACTGGGTGCTTGATAGGC/AACCAAAATATCCGGAGTAAAAGA	164	1.90	[56]	GU585871
*SAND*	CAACATCCTTTACCCATTGACAGA/GCATTTGATCCACTTGCAGATAAG	76	1.88	[56]	GU585872
*NAD5*	GATGCTTCTTGGGGCTTCTTGTT/CTCCAGTCACCAACATTGGCATAA	181	1.82	[83]	GU585873
**b) Candidate genes**					
*PAL*	TCTGGTGGAAGGAATCCAAG/CAAAGTGCCACCAGGTAGGT	230	1.77	[62]	GU585850
*CHS1*	AGCCAGTGAAGCAGGTAGCC/GTGATCCGGAAGTAGTAAT	155	1.74	[61]	GU585851
*CHS2*	TCTGAGCGAGTATGGGAACA/AGGGTAGCTGCGTAGGTTGG	294	1.80	[61]	GU585852
*CHS3*	TCACTTGGACAGCCTTGTTG/CAATTCGAACATGGGCTTCT	106	1.87	$	GU585853
*CHI1*	CAGGCAACTCCATTCTTTTC/TTCTCTATCACTGCATTCCC	103	1.69	[84]	GU585854
*CHI2*	TCCAGATCAAGTTCACAGCA/GAAACAAGAGCCTCAAAGAA	127	1.60	[84]	GU585855
*F3'H*	ATTCGCCACCCTGAAATGAT/AGCCGTTGATCTCACAGCTC	196	1.82	[15]	GU585856
*F3'5'H*	GAAGTTCGACTGGTTATTAACAAAGAT/ AGGAGGAGTGCTTTAATGTTGGTA	156	1.68	[15]	GU585857
*F3H1*	CCAATCATAGCAGACTGTCC/TCAGAGGATACACGGTTGCC	69	1.83	[84]	GU585858
*F3H2*	CTGTGGTGAACTCCGACTGC/CAAATGTTATGGGCTCCTCC	129	1.70	[84]	GU585859
*DFR*	GAAACCTGTAGATGGCAAGA/GGCCAAATCAAACTACCAGA	114	1.85	[84]	GU585860
*LDOX*	AGGGAAGGGAAAACAAGTAG/ACTCTTTGGGGATTGACTGG	109	1.76	[84]	GU585861
*UFGT*	GGGATGGTAATGGCTGTGG/ACATGGGTGGAGAGTGAGTT	152	1.74	[84]	GU585862
*MybA1*	TAGTCACCACTTCAAAAAGG/GAATGTGTTTGGGGTTTATC	66	1.67	[84]	GU585863
*FLS1*	CAGGGCTTGCAGGTTTTTAG/GGGTCTTCTCCTTGTTCACG	154	1.82	[85]	GU585864
*LAR1*	AAATGAACTCGCATCTGTGT/CTGTGGGATGATGTTTTCTC	109	1.75	[82]	GU585865
*LAR2*	TGATATCAGCTGTGGGTGGA/CCCAAATTCTGATGGAAGGA	104	1.74	$	GU585866
*ANR*	GCTGCTGTTACCATCAATCA/GCAGGATAGCCCCAAGTAGG	113	1.62	[82]	GU585867

^1^See legends for Figure 1 and Figure 3 for names of genes

*Accession numbers indicate sequences generated from this study.

^$^Primers were designed based on sequence available in GenBank.

### Expression stability analysis of candidate reference genes

**Figure 3:**
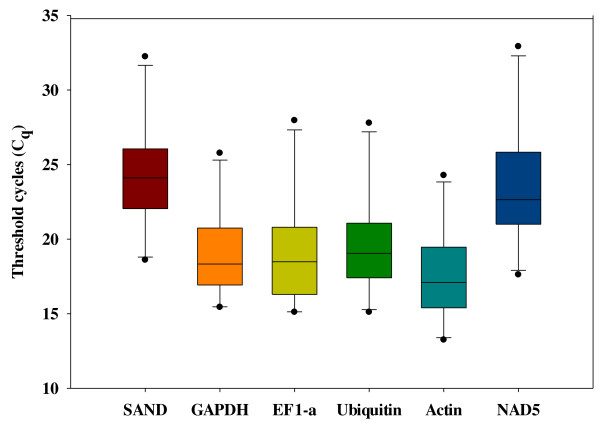
**Box plot representation of raw C_q _values obtained from amplification curves for reference genes**. Lower and upper boundaries of each box indicate the 25^th ^and the 75^th ^percentile, respectively. Ranges are represented as bars (whiskers) below and above the box and indicate the 10^th ^and 90^th ^percentiles, respectively. The horizontal line in each box represents mean and outliers by (•). *SAND*: SAND family protein; *GAPDH*: glyceraldehyde 3-phosphate dehydrogenase; *EF1-a*: elongation factor1-alpha; *Ubiquitin*: ubiquitin-60S ribosomal L40 fusion protein; *Actin*, *NAD5*: NADH dehydrogenase subunit 5.

A total of six putative reference genes (Table 2) were evaluated for their expression stability under our experimental conditions. Since all RT-qPCR reactions were performed with cDNA derived from equal quantity of total RNA, transcript abundance of these six genes were analyzed by direct comparison of C_q _values, assuming equal C_q _for equal transcript number. As shown in Figure 3, the six reference genes were grouped into two arbitrary categories based on their C_q _values combined from both GLRaV-3-infected symptomatic and virus-free green leaf samples. Four genes (*GAPDH*, *EF1-a*, *Ubiquitin *and *Actin*) showed higher transcript levels, since they presented C_q _values with a median between 15 and 20 cycles. The other two (*SAND *and *NAD5*) were categorized as genes with relatively low transcript levels, since their median C_q _values were between 20 and 25 cycles.

**Figure 4:**
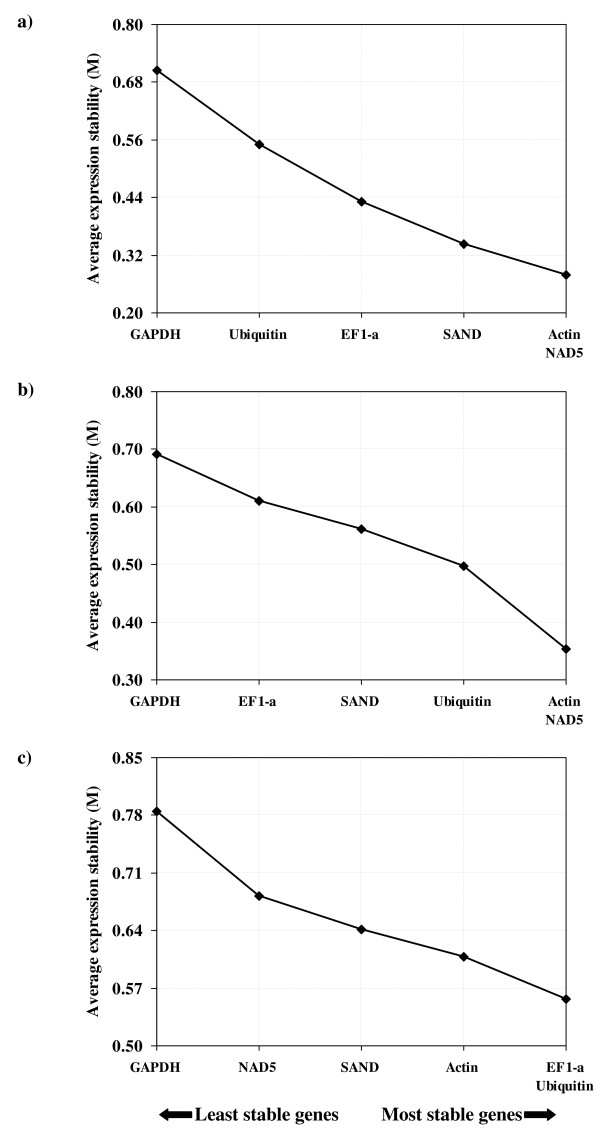
**Stability of reference genes in grapevine leaves**. Stability value (M) for a set of reference genes is analyzed with *geNorm *algorithm in (a) GLRaV-3-infected (designated as virus-infected), (b) virus-free and (c) combined (virus-free and virus-infected) samples. Reference genes in the x-axis are ranked from left to right based on average expression stability. The *GAPDH *gene in the extreme left in all graphs with the highest M value denotes lowest expression stability among the reference genes in all samples. Genes at the extreme right in each graph shows the highest expression stability among the reference genes. See legend for Figure 3 for names of reference genes.

The raw C_q _data for each reference gene was subsequently analyzed using *geNorm *algorithm to evaluate their expression stability in virus-infected and virus-free samples and ranked according to their expression stability measure "M" (Figure 4a-c). All six genes showed high expression stability and had M values lower than 0.8, below the default limit of 1.5 suggested by the *geNorm *program. From this analysis, *Actin *and *NAD5 *genes were estimated to have the lowest M values, indicating that these two genes showed high expression stability in both virus-infected and virus-free samples (Figure 4a&4b). *GAPDH *gene gave the highest M value and hence considered as having lowest stability in both types of samples under our experimental conditions. However, the M values for *SAND*, *EF1-a *and *Ubiquitin *were variable between the two types of samples, indicating differences in their expression stability due to virus infection. The expression stability of the six reference genes differed when M values were calculated by combining raw C_q _data of each gene from both virus-infected and virus-free samples (Figure 4c). In this case, *EF1-a *and *Ubiquitin *were estimated to have the lowest M values, followed by *Actin*, *SAND *and *NAD5 *genes. Based on these results, a subset of two genes (*Actin *and *NAD5*) was used to calculate normalization factor (NF) through the geometrical averaging of their raw C_q _values. The resulting NF was used to normalize raw C_q _data generated in RT-qPCR for flavonoid biosynthetic pathway genes in virus-infected and virus-free grapevine leaves.

### Expression patterns of flavonoid biosynthetic pathway genes

**Figure 5:**
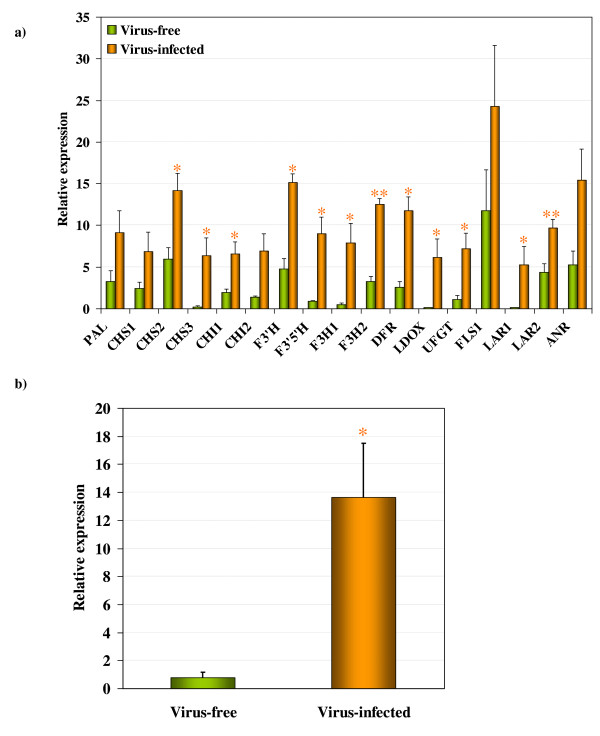
**Expression patterns of flavonoid biosynthetic pathway genes in GLRaV-3-infected symptomatic and virus-free green leaves**. The relative expression levels of (a) the flavonoid biosynthetic pathway genes and (b) the *MybA1 *gene in GLRaV-3-infected (designated as virus-infected) and virus-free leaves are shown as arbitrary units on the y-axis. The raw C_q _values for each gene was normalized using two reference genes (NF_[*Actin *and *NAD5*]_). Columns represent mean value from five biological replicates, except in case of *MybA1 *that represents only four biological replicates and vertical bars indicate standard errors. Significant differences between virus-infected and virus-free leaves was determined by one-way ANOVA, using the SigmaPlot 11 software and indicated by asterisks (* = *p *< 0.05 and ** = *p *< 0.001). See legend for Figure 1 for names of genes.

The expression patterns of flavonoid upstream pathway gene (*PAL*), genes involved in the biosynthesis of different flavonoids (*CHS1, CHS2, CHS3, CHI1, CHI2*, *F3'H*, *F3'5'H*, *F3H1, F3H2*, *DFR*, *LDOX*, *UFGT, FLS1*, *LAR1, LAR2 *and *ANR*) and a regulatory gene (*MybA1*) were examined in GLRaV-3-infected symptomatic and virus-free green leaves. The distribution overview of expression levels of gene transcripts showed (Additional file 3, Figure S3) that many of the flavonoid biosynthetic pathway genes from virus-infected samples presented lower median C_q _values. This indicated higher transcript levels for these genes in virus-infected symptomatic leaves, assuming equal C_q _for equal transcript number, since all RT-qPCR reactions were performed with equal amount of cDNA derived from equal quantity of total RNA. Using the two best reference genes identified (NF_[*Actin *and *NAD5*]_) from gene expression stability analyses described above, we normalized the raw C_q _data for each gene from virus-infected and virus-free samples and their relative expression levels are shown in Figure 5a&5b. In general, flavonoid biosynthetic pathway genes analyzed in this study showed higher expression levels in virus-infected symptomatic leaves when compared with expression levels of corresponding genes from virus-free green leaves. The expression levels of these genes as fold increase in virus-infected symptomatic leaves over the corresponding values from virus-free green leaves is shown in Table 3. Their expression levels ranged from two- to fifty-fold increase in virus-infected samples. Among them, *CHS3*, *F3'5'H*, *F3H1*, *LDOX *and *LAR1 *showed greater than 10-fold increase suggesting that these genes were expressed at higher levels in virus-infected leaves. *MybA1*, which regulates anthocyanin biosynthesis in grapevines via expression of the *UFGT *gene, was expressed by about 19-fold higher in virus-infected symptomatic than in virus-free green leaves (Figure 5b, Table 3). Similar trend in expression levels of flavonoid biosynthetic pathway genes and *MybA1 *was obtained when the two best reference genes (NF_[*EF1-a *and *Ubiquitin*]_), identified when expression stability of reference genes was calculated by combining raw C_q _data from both virus-infected and virus-free samples (Figure 4c), were considered for data normalization (Table 3). However, the values were slightly lower than those obtained when *Actin *and *NAD5 *were used as reference genes for data normalization. Based on these results, it can be concluded that some of the flavonoid biosynthetic pathway genes are significantly up-regulated in virus-infected symptomatic leaves when compared to expression levels of corresponding genes in virus-free green leaves.

**Table 3:** Relative fold increase of flavonoid biosynthetic pathway genes in GLRaV-3-infected, symptomatic leaves over virus-free green leaves

Gene^1^	*Actin *+ NAD5*	*EF1-a *+ Ubiquitin*
*PAL*	4.99 ± 2.99	3.00 ± 1.36
*CHS1*	4.23 ± 1.34	2.93 ± 0.65
*CHS2*	3.02 ± 0.97	2.05 ± 0.41
*CHS3*	37.43 ± 5.09	28.91 ± 6.97
*CHI1*	3.42 ± 0.24	2.62 ± 0.52
*CHI2*	4.40 ± 1.35	3.40 ± 1.24
*F3'H*	4.60 ± 1.69	3.14 ± 0.80
*F3'5'H*	11.33 ± 2.91	8.52 ± 2.62
*F3H1*	23.62 ± 11.47	15.45 ± 5.46
*F3H2*	4.83 ± 1.30	3.32 ± 0.62
*DFR*	5.73 ± 1.13	3.82 ± 0.41
*LDOX*	40.75 ± 15.59	25.56 ± 6.36
*UFGT*	9.22 ± 1.53	6.77 ± 1.11
*MybA1*	19.03 ± 5.56	12.04 ± 1.92
*FLS1*	3.77 ± 1.33	2.54 ± 0.70
*LAR1*	58.35 ± 18.54	36.90 ± 6.91
*LAR2*	2.51 ± 0.29	1.82 ± 0.28
*ANR*	3.84 ± 1.14	3.18 ± 1.50

^1^See legend for Figure 1 for names of genes

*Values are mean ± SE.

Among the three isogenes of chalcone synthase (*CHS1*, *CHS2 *and *CHS3*) that are involved in recruitment of flavonoid precursors to enter the flavonoid biosynthetic pathway, *CHS1 *and *CHS2 *showed about 4- and 3-folds higher expression levels, respectively, while *CHS3 *exhibited about 37-fold increase in virus-infected leaves. These results indicate preferential up-regulation of *CHS3 *in virus-infected symptomatic leaves when compared with virus-free green leaves. The two flavonoid hydroxylases, *F3'H*, which regulates the synthesis of cyanidin-based anthocyanins, and *F3'5'H*, which regulates the synthesis of delphinidin-based anthocyanins, were expressed at about 5- and 11-fold higher, respectively, in virus-infected symptomatic leaves compared to virus-free green leaves. Higher expression levels of the flavonoid pathway genes like *F3H1 (~23-fold)*, *DFR (~6-fold)*, *LDOX (~40-fold)*, and *UFGT (~9-fold) *and *LAR1 *(~*58-fold*) genes specific to anthocyanins and proanthocyanidins, respectively, in virus-infected symptomatic leaves indicate enhanced synthesis of anthocyanins and proanthocyanidins in these leaves. It is likely that the synthesis of more flavonols was also favored in virus-infected leaves due to ~4-fold higher expression levels of the *FLS1 *gene. Higher expression levels of *LAR1*, *LAR2 *and *ANR *indicate that these genes were contributing to the enhanced synthesis of proanthocyanidins in virus-infected leaves.

### Estimation of anthocyanins, flavonols and proanthocyanidins

**Figure 6:**
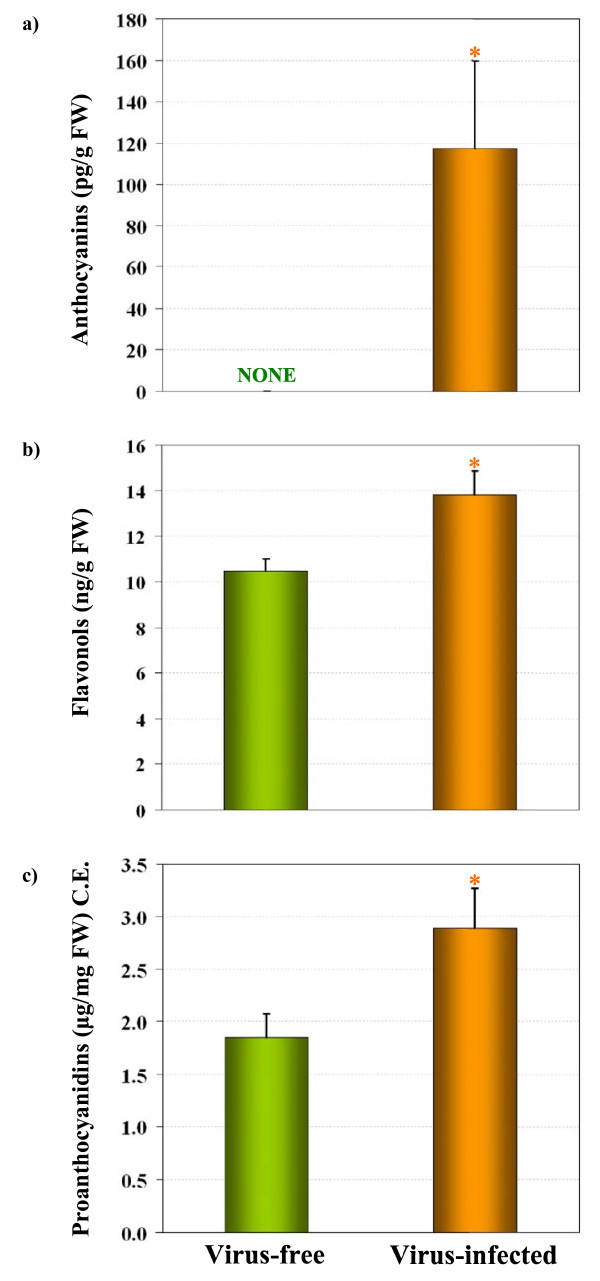
**Estimation of flavonoids in GLRaV-3-infected symptomatic and virus-free green leaves**. Total amounts of (a) anthocyanins, (b) flavonols and (c) proanthocyanidins from GLRaV-3-infected (designated as virus-infected) and virus-free samples are shown. Columns represent mean value from five biological replicates and vertical bars indicate standard errors. NONE in (a) indicates no anthocyanins detected in virus-free leaves. Significant differences between virus-infected and virus-free leaves were determined by one-way ANOVA using the SigmaPlot 11 software and indicated by asterisks (* = *p *< 0.05). C.E. = catechin equivalent.

**Figure 7:**
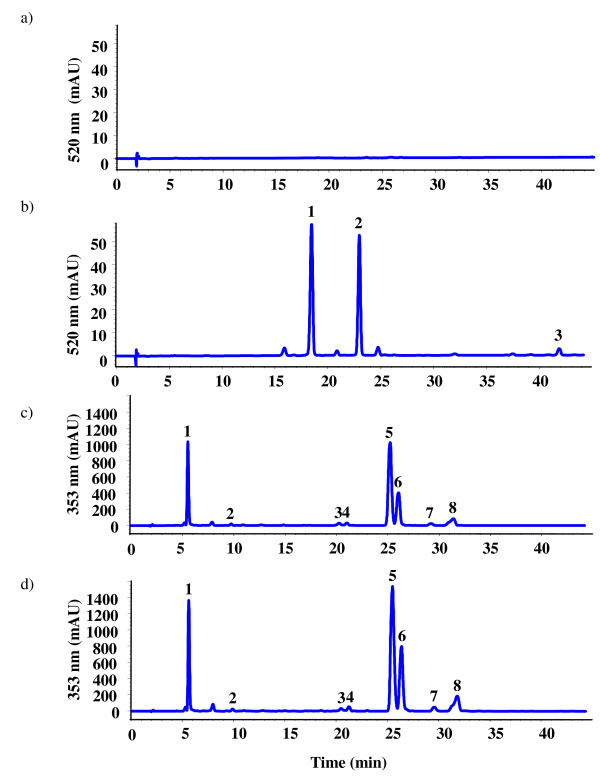
**HPLC profiling of anthocyanins and flavonols in GLRaV-3-infected symptomatic and virus-free green leaves**. The chromatograms show profile of anthocyanins from (a) virus-free and (b) GLRaV-3-infected leaves and profile of flavonols from (c) virus-free and (d) virus-infected leaves. None in (a) indicates no anthocyanins detected in virus-free, green leaves. Anthocyanins identified in (b) are: 1 = Cyanidin-3-glucoside; 2 = Malvidin-3-glucoside; 3 = Peonidin-3-O-6-coumarilated. Flavonols identified in (c) and (d) are: 1 = Myricetin-3-glucoside; 2 = Unknown; 3 = Unknown; 4 = Unknown; 5 = Quercetin-3-glucoside; 6 = Quercetin-3-glucuronide; 7 = Unknown; 8 = Unknown.

To be able to correlate gene expression data with the accumulation of different flavonoid compounds, we analyzed the secondary metabolite constituents of GLRaV-3-infected symptomatic and virus-free green leaves. Anthocyanins and flavonols were analyzed by HPLC and proanthocyanidins were estimated by protein precipitation method as total tannins. Figure 6 shows total amounts of anthocyanins, flavonols and proanthocyanidins and Figure 7 shows HPLC profiles of anthocyanins and flavonols from virus-infected and virus-free leaves. Anthocyanins were not detected in virus-free green leaves (Figure 6a and 7a), whereas two clearly discernible peaks (numbered 1 and 2 with increasing retention times) were observed in virus-infected leaves (Figure 7b) with no corresponding peaks in virus-free green leaf samples. Based on their retention times and spectral data, the two major peaks in virus-infected leaves were identified as cyanidin-3-glucoside and malvidin-3-glucoside. Further analysis indicated that cyanidin-3-glucoside accounted for 61% and malvidin-3-glucoside accounted for 39% of total anthocyanins detected in virus-infected leaves. A minor peak, designated as #3 in Figure 7b, was tentatively identified as Peonidin-3-O-6-coumarilated. Although total flavonols were detected in both virus-infected and virus-free leaves (Figure 6b), they were 24% higher in virus-infected leaves than in virus-free leaves. As shown in Figure 7c&7d, HPLC analysis showed three clear peaks in both virus-infected and virus-free leaves. Based on retention times, they corresponded to putative myricetin (peak 1) and quercetin (peak 5 & 6) derivatives, respectively, with quercetin derivatives accounting for about 69% and myricetin derivatives accounting for about 20% of total flavonols and the rest accounting for other unidentified flavonols. Estimation of proanthocyanidins in leaves as total tannins showed that their concentration was 36% higher in virus-infected than virus-free leaves (Figure 6c). It is important to note that the method we used to measure proanthocyanidins is limited to estimating total amount of these compounds rather than a method that provides structural information. Potentially both delphinidin and cyandin sub-units are present in both types of leaf tissues. Taken together, the above results (Figure 6) indicated that the three classes of flavonoids (anthocyanins, flavonols and proanthocyanidins) are present in significantly higher amounts in virus-infected leaves and correlate with up-regulation of the flavonoid biosynthetic pathway genes shown in Figure 5 and Table 3.

## Discussion

The necessity for ensuring quality-assurance measures in RT-qPCR analysis of gene expression is well recognized and a set of guidelines have been outlined for appropriate normalization strategy to control for non-specific variation between samples [49]. Although a range of endogenous reference genes have been listed as good candidates for normalization of gene expression, identification of the most suitable reference genes for the given experimental conditions, rather than using reference genes published in the literature, is extremely important in functional genomics studies [47,48]. In addition, certain reference genes may be stably expressed in one plant species but are not be well suited for use in other species [50]. Apart from other fields of research, this knowledge is highly relevant to studies in plant host-virus interactions, as viruses are known to modulate key cellular processes in plants which may involve changes in the expression of endogenous host genes normally used as reference genes in RT-qPCR [51,52]. Moreover, viruses manipulate different host cellular transcription pathways and the extent to which these pathways are affected will be dependent on the specific virus-host combination [53,54]. Consequently, we evaluated geometric averaging of multiple reference genes as a means to avoid experimental bias in gene expression data.

In this study, we analyzed a set of six putative reference genes (*Ubiquitin, Actin*, *GAPDH*, *EF1-a, SAND *and *NAD5*) for their expression stability in leaf samples collected from a red-fruited wine grape cultivar (cv. Merlot) grown under field-conditions. Since expression stability of reference genes is known to vary with environmental conditions under which plants are grown, the type of plant tissue used and under a diverse set of biotic and abiotic stress conditions, we validated the expression stability of these six genes under our experimental conditions using the *geNorm *software and selected *Actin *and *NAD5 *to normalize RT-qPCR data obtained for the flavonoid biosynthetic pathway genes in virus-infected and virus-free grapevine leaves [55]. In a previous study, *GAPDH *was ranked as one of the top three reference genes (*GAPDH *<*Actin *<*EF1-a*/*SAND*) for gene expression studies in grape berry development [56]. However, we found that *GAPDH *is the least reliable in the context of our investigations on relative expression of the flavonoid biosynthetic pathway genes in grapevine leaf samples (Figure 4). These results clearly highlight the importance of validating reference genes as the most invariant internal controls for a particular experimental condition prior to investigating the relative expression of target genes by RT-qPCR.

By using gene-specific RT-qPCR, we present evidence in this study, the first example to our knowledge, that overall up-regulation of *PAL*, an enzyme that commits the flux of primary metabolism into the flavonoid biosynthetic pathway, and both "early" (*CHS*, *CHI*, *F3'H*, *F3'5'H*, *F3H *and *FLS*) and "late" genes (*DFR, LDOX, UFGT *and *LAR*) of the pathway occurred in GLRaV-3-infected symptomatic grapevine leaves (Figure 5). In red-fruited cultivars of wine grapes, anthocyanin pigments accumulate predominantly in berry skins displaying various shades of colors ranging from brick red to dark blue and their biosynthesis is developmentally triggered at the onset of *véraison *via the activation of flavonoid biosynthetic pathway genes [25]. Under normal circumstances, these cultivars do not exhibit such coloration in their foliage during the growing season. Thus, changes in leaf color (Figure 2) and accumulation of specific classes of anthocyanins (Figure 6 and 7) only in GLRaV-3-infected symptomatic leaves supported our hypothesis that expression of the flavonoid biosynthetic pathway genes was activated in virus-infected leaves. Although this study was based on the expression analysis of flavonoid biosynthetic pathway genes and qualitative and quantitative variation of anthocyanins, flavonols and proanthocyanidins, it should be noted that mRNA expression is only one aspect of functional gene regulation of the pathway that result in changes in color of leaves in virus-infected plants. Since changes in leaf coloration begins to occur soon after *véraison*, even though GLRaV-3 can be detected in leaves of infected grapevines during the entire season including pre-*véraison*, it remains to be studied if the specific induction of anthocyanins in virus-infected leaves during post-*véraison *is tightly coupled with a cascade of physiological and/or molecular events triggered as a consequence of virus-host interactions during *véraison*.

In plants, delphinidin- and cyanidin-based anthocyanins exhibit blue and reddish color, respectively, under the acidic conditions of plant vacuoles [17]. HPLC profiling of total anthocyanins showed that both cyanidin-3-glucoside and malvidin-3-glucoside accumulated in virus-infected symptomatic leaves and they are virtually undetected in virus-free green leaves (Figure 6a and Figure 7a&7b). We believe that presence of these two classes of anthocyanins, although cyanidin-3-glucoside is slightly but not significantly higher than malvidin-3-glucoside in virus-infected leaves, contributes to red and reddish-purple discoloration of virus-infected leaves. Since *F3'5'H *regulates the synthesis of delphinidin-based anthocyanins and *F3'H *regulates the synthesis of cyanidin-based anthocyanins, expression profiles of these two genes in concert with increased expression of anthocyanin-specific gene *UFGT *and its transcription factor gene *MybA1 *would ensure the flux of flavonoid intermediates towards the synthesis of these two classes of anthocyanins in virus-infected leaves. The levels of *F3'H *and *F3'5'H *gene transcripts observed in virus-free green leaves is in agreement with recent reports that *F3'H *gene was only slightly detectable and *F3'5'H *gene was expressed at non-detectable levels in green, fully expanded grapevine leaves [15,57]. Our results also showed significantly higher levels of flavonols in virus-infected leaves than in virus-free leaves, and the predominant flavonols were quercetin followed by myricetin (Figure 6b and 7c&7d). Bogs *et al*. showed that total amounts of proanthocyanidins decline with leaf maturity and the two LAR isogenes have different patterns of expression with *LAR1 *showing seed-specific expression and insignificant levels in mature leaves and *LAR2 *readily present in different tissues, including leaves [58]. Hummer and Schreier reported that proanthocyanidins as condensed tannins can precipitate proteins and several methods using protein precipitation have been used to estimate proanthocyanidins in various agricultural products [59]. Using this approach, we showed that higher amounts of proanthocyanidins are present in virus-infected leaves than in virus-free leaves (Figure 6c) and the data correlate with strong induction of proanthocyanidin-specific genes; namely, *LAR1*, *LAR2 *and *ANR*. Since *LAR *and *ANR *genes provide two separate pathways for the synthesis of the terminal units of proanthocyanidin polymers, specific induction of *LAR1 *in virus-infected leaves (Figure 5a) would suggest that this gene may be contributing towards higher amounts of proanthocyanidins [58]. Overall, these results are compatible with our hypothesis that activation of the flavonoid biosynthetic pathway genes occurred in GLRaV-3-infected symptomatic leaves during post-*véraison *period resulting in *de novo *synthesis of specific flavonoid classes and leading to phenotypic expression of GLRD symptoms. It is also likely that these flavonoid compounds confer protection from oxidative damage and/or against attack by opportunistic pathogens due to their antioxidant and free radical scavenging properties [8,9,60].

The use of more sensitive and gene-specific RT-qPCR technique enabled us to study relative abundance of the three highly homologous CHS gene family transcripts in virus-infected grapevine leaves. The results showed that three members of the CHS family (*CHS1*, *CHS2 *and *CHS3*) identified to date in grapevine, accumulated to varying levels, with *CHS3 *expression being significantly higher than the other two isogenes indicating its important role in color development in virus-infected leaves. This result is consistent with previous studies that *CHS3*, which is phylogenetically divergent from a cluster formed together by *CHS1 *and *CHS2*, was predominant in grape berry skins of red-fruited cultivars during coloration [61,62]. The exact role of *CHS1 *and *CHS2 *in the biosynthesis of flavonoids may be insignificant, although their expression was implicated in the production of proanthocyanidins in unpigmented tissues of both red- and white-fruited grapevine cultivars [61,62]. Among the two flavanone-3-hydroxylase isogenes, *F3H1 *showed higher expression levels than *F3H2*, and *LAR1 *of the two LAR isogenes of leucoanthocyanidin reductase was expressed at higher levels in virus-infected leaves. No such differential expression was observed in CHI isogenes. Thus, members of multigenic families appear to be induced differentially during the biosynthesis of flavonoids in virus-infected leaves of cv. Merlot showing GLRD symptoms.

Induced accumulation of anthocyanins and development of reddish-purple coloration in GLRaV-3 infected grapevine leaves appears to be analogous in some ways with stimulation of pigmentation in other plant species infected with taxonomically disparate viruses [63]. It has been shown that mottling symptoms present on the seed coats of virus-infected soybean plants or induction of floral anthocyanin pigmentation in petunias can be caused by suppression of CHS posttranscriptional gene silencing (PTGS) via the expression of a virus-encoded silencing suppressor protein and that the reversion to pigmentation in virus-infected tissues is correlated with an increase in the CHS mRNA level [64-66]. Since CHS is the first committed enzyme in the flavonoid biosynthetic pathway, it is tempting to speculate that modulation of PTGS suppression of CHS isogenes by GLRaV-3-encoded silencing suppressor protein(s) occurs during post-*véraison *in virus-infected grapevine leaves leading to a cascade of molecular events resulting in up-regulation of *CHS3 *and the ensuing production of secondary metabolites conferring color to otherwise green leaves. However, identification of silencing suppressors of GLRaV-3 awaits further validation of this possibility.

An alternative explanation would be that, since grapevine leaves begin to show GLRD symptoms only during post-*véraison *even though GLRaV-3 can be detected in infected plants throughout the season (i.e. both during pre- and post-*véraison*) and the virus is phloem-limited, appearance of reddish-purple coloration in symptomatic leaves could be due to a consequence of changes occurring in host metabolism and altered phloem translocation during *véraison*. In this context, up-regulation of the flavonoid biosynthetic pathway genes in GLRaV-3-infected Merlot leaves may not entirely represent a host defense response to pathogen infection and, therefore, our results differ somewhat from other compatible plant-pathogen interactions in grapevine leaves and hybrid poplar, where genes encoding key enzymes of the flavonoid biosynthetic pathway were strongly induced after infection with phytoplasma or fungal pathogens [67-70]. Nevertheless, the present study contributes towards a better understanding of virus-host interactions leading to the development of GLRD symptoms in red-fruited wine grape cultivars.

In the present study, we observed higher transcript levels of *MybA1 *gene that encodes a MYB transcription factor in virus-infected leaves (Figure 5b). Although other MYB transcription factors have recently been reported in grapevines, our rationale for analyzing only *MybA1 *was because of its main role in the regulation of anthocyanin biosynthesis via expression of the *UFGT *gene [20,71]. However, further research is necessary to determine whether fine regulation of the flavonoid biosynthetic pathway genes in virus-infected leaves involves a combinatorial action(s) of different R2R3-MYB transcription factors, including basic helix-loop-helix (bHLH) and WD40 factors expressed in a spatially and temporally controlled manner [3,72].

It has been documented that the flavonoid biosynthetic pathway in fruits and vegetative tissues of plants is up-regulated by different environmental stress factors and in response to nutritional status [73]. It has also been suggested that in woody perennials like red-osier dogwood, anthocyanins accumulate during senescence to provide optical masking of chlorophyll in order to reduce the risk of photo-oxidative damage to leaf cells [74]. However, reduced levels of chlorophylls and carotenoids and higher amounts of specific classes of anthocyanins and the resulting changes in coloration of GLRaV-3-infected grapevine leaves during post-*véraison *may represent specific host-virus interactions as discussed above rather than a generalized abiotic stress response to environmental and/or nutritional imbalances. An integrated approach involving proteomic and metabolomic analyses combined with studies on modulation of cellular transcriptome would provide additional data for a comprehensive understanding of events that underlie changing colors of virus-infected grapevine leaves in red-fruited cultivars during post-*véraison *stage of berry development. Such information would also help to delineate grapevine's response to compatible virus infections from generic stress responses stimulated by a variety of abiotic and environmental factors.

Since berries in many red-fruited wine grape cultivars infected with GLRD show uneven ripening with reduced levels of extractable anthocyanins from berry skins (Naidu *et al*., unpublished results), the methodologies and results described in this study is providing leads for a deeper exploration of impacts of GLRD on berry skin pigments at the molecular level. In addition, there are several outstanding questions in GLRD-grapevine interactions that need to be addressed. They include: Do other red-fruited wine grape cultivars exhibit similar responses in the expression of flavonoid biosynthetic pathway genes and the profile of flavonoids to infection with GLRaV-3? Do genetically different GLRaVs trigger homologous responses in different red-fruited wine grape cultivars? Is the absence of dramatic symptoms in white-fruited wine grape cultivars an indication of non-responsiveness of the flavonoid biosynthetic pathway to virus infection? Indeed, GLRD-grapevine offers an excellent model system to address these questions.

## Conclusions

In summary, we compared the relative expression of the flavonoid biosynthetic pathway genes between GLRaV-3-infected symptomatic and virus-free green leaves in a red-fruited wine grape cultivar (cv. Merlot) using RT-qPCR. The results showed up-regulation of genes in virus-infected symptomatic leaves suggesting modulation of the pathway towards *de novo *synthesis of certain classes of end-products and laid a foundation for deeper exploration of molecular mechanisms of biosynthesis and accumulation of flavonoids in virus-infected wine grape cultivars. The information on evaluation of reference genes suggests that validation of a set of reference genes as the most invariant internal controls for a particular experimental condition is essential for exploring genomics of plant-virus interactions in ecologically relevant, agriculturally important non-model perennial crops like grapevine under field conditions.

## Methods

### Plant samples

Leaf samples used in this study came from 10 year-old, own-rooted grapevines (cv. Merlot). The block is located near Prosser in Washington State, USA (46.2°N latitude, 119.8°W longitude), and the grapevines are grown under standard viticultural practices with drip irrigation. The grapevines were spaced 6 ft within rows and 8 ft between rows and the rows are in North-South orientation. The vineyard soil was classified as sandy loam. Plants for sampling were selected in such a way that individual grapevines exhibiting typical GLRD symptoms are adjacent to disease-free grapevines in a given row to minimize error in sampling and experimental results due to variations in growing conditions. Each pair of symptomatic and adjacent non-symptomatic grapevines was tested for different grapevine viruses by RT-PCR [75]. Mature leaves at the 4^th ^and 5^th ^node from the basal portion of primary canes showing typical symptoms of GLRD from virus-infected vines and comparable leaves from adjacent virus-free vines (Figure 2) were collected at the same time in mid September (representing post-*véraison *stage of berry development) to minimize variation due to developmental stage of leaves. The leaves were frozen immediately in liquid N_2 _upon collection in the field, transported to the lab in liquid N_2 _and stored at -80°C until required for RNA extraction. Leaves from individual grapevines were pooled and a pair of adjacent virus-infected and virus-free grapevines constituted one biological replicate. A total of five biological replicates (i.e. five virus-infected and five virus-free grapevines) were used for this study. Anecdotal evidence suggested that GLRD was introduced into the vineyard block via planting virus-infected cuttings. Hence, there is no bias in the age of virus-infected and virus-free grapevines used in this study.

### Estimation of chlorophylls and carotenoids

Frozen leaf tissue (100 mg) was extracted in 80% acetone and total chlorophylls and carotenoids were estimated using a spectrophotometer [76,77]. Leaves from five virus-infected grapevines along with their respective controls were used separately and pigments estimated by two independent times using separate batches of tissue.

### RNA isolation

Total RNA was isolated from leaves using Spectrum Plant Total RNA kit (Sigma-Aldrich, St Louis, MO, USA) following the manufacturer's instructions. Any contaminating genomic DNA was removed by on-column DNase I digestion (Qiagen Inc., Valencia, CA, USA). The integrity of RNA was verified by resolving in 1% formaldehyde-agarose gels and subsequent ethidium bromide staining. RNA purity was assessed based on absorbance ratio of 1.8 to 2.0 at 260/280 nm using Nanodrop ND-1000 spectrophotometer (NanoDrop Technologies, Rockland, DE, USA).

### Primers, RT-PCR and analysis of gene sequences

Sequences of primers used in this study were retrieved from literature and used for amplifying partial gene-specific sequences. A list of primer pairs and amplicon lengths are provided in Table 2. One μg of total RNA was reverse transcribed in 25 μl reaction mixture containing gene-specific complementary primer using Superscript III reverse transcriptase kit (Roche Diagnostics, Mannheim, Germany) by following the manufacturer's instructions. Reverse transcription (RT) was carried out at 50°C for 30 min followed by thirty five consecutive cycles of PCR amplification (denaturation at 94°C for 30 s, annealing at 56°C for 30 s, extension at 72°C for 30 s), with a final extension at 72°C for 5 min using 1 μM each of gene-specific forward and reverse primers. Amplified fragments specific to each gene were cloned separately into pCR 2.1-TOPO vector (Invitorgen, Carlsbad, CA) and recombinant clones purified using QIAGEN plasmid mini-prep kit (Qiagen Inc., Valencia, CA, USA). Two independent clones were sequenced in both orientations by automated DNA sequencing at Molecular Biology Core facility at the Center for Reproductive Biology, Washington State University, Pullman, WA, USA. The sequences were compared with corresponding sequences in GenBank with BLAST 2 sequences software (http://www.ncbi.nlm.nih.gov/Blast.cgi). The partial sequences of genes obtained in this study were deposited in GenBank with accession numbers GU585850 to GU585873.

### Reverse transcription-quantitative real-time PCR

One μg of total RNA was reverse transcribed in 20 μl reaction mixture containing oligo d(T)_18 _primer using the Transcriptor First Strand cDNA Synthesis Kit (Roche Diagnostics, Mannheim, Germany) by following the manufacturer's instructions. Quantitative real-time PCR (qPCR) reactions were performed in 384-well plates with LightCycler^® ^480 real-time PCR instrument (Roche Diagnostics, Mannheim, Germany) using SYBR Green I Master Mix (Roche Diagnostics, Mannheim, Germany) as described in the manufacturer's manual. All qPCR assays were performed with proper controls according to Minimum Information for Publication of Quantitative Real-Time PCR Experiments (MIQE) guidelines [49]. Each reaction was carried out in 20 μl reaction mixture containing 2 μl of cDNA, 0.5 μM each of gene-specific forward and reverse primer (Table 2) and 10 μl of 2 × SYBR Green I Master Mix (Roche Diagnostics, Mannheim, Germany). The following conditions were used for each qPCR assay: denaturation for 5 min at 95°C, followed by 45 cycles of PCR (10 s at 95°C for template denaturation, 10 s at 56°C for annealing and 30 s at 72°C for extension). All assays included no-RT and no-template controls to verify non-specific amplification. At the end of each qPCR, a melting curve analysis was performed over the range 65-97°C to determine the specificity of amplicons (Additional file 2, Figure S2). The amplicons were also resolved in 1.2% agarose gels, stained with ethidium bromide and visualized under UV light. cDNA from five biological replicates (virus-infected and virus-free leaves collected from five individual grapevines for each category) were used for qPCR analysis, and three technical replicates were analyzed for each biological replicate. Aliquots from the same cDNA were used in all technical replications.

LightCycler^® ^480 Software (version 1.5; Roche Diagnostics) was used to analyze the data. We used the term *quantification cycle *(C_q_), instead of *threshold cycle *(C_t_), *crossing point *(C_p_) or *take-off point *(TOP) currently used in the literature, to describe the fractional qPCR cycle used for quantification according to the Real-Time PCR Data Markup Language (RDML) data standard [78]. The C_q _is defined as the number of cycles at which the fluorescence signal exceeds a specific threshold level of detection and is inversely correlated with the amount of target nucleic acid present in the reaction. qPCR efficiencies (E) were calculated using the equation E = 10^-1/slope ^on a standard curve generated based on 10-fold dilution of gene-specific plasmid DNA (five dilution points, starting with 10 pg of respective plasmid DNA of each gene). The LightCycler^® ^480 Software automatically calculates the efficiency and displays it on the analysis window.

### Expression stability analysis of reference genes

Six candidate reference genes were selected for this study (Table 1). Reference gene stability analyses were performed with the Microsoft excel-based *geNorm *software program available at http://medgen.ugent.be/genorm/[43]. The *geNorm *software uses pairwise comparison method to calculate gene expression stability measure "M" for a potential reference gene in a given cDNA sample panel. This measure was demonstrated in many studies to be valuable for selecting appropriate reference genes across several experimental conditions and treatments [45]. Using this program, the average expression stability value M (defined as the constancy of the expression ratio between two reference genes across samples) for each gene was obtained in a stepwise fashion excluding the gene with the highest M for the next calculation round. This process was repeated until only two genes remained. Genes with an M value below the default limit of M = 1.5 were considered as having acceptable expression stability (or suitability as normalizing gene) and genes with the lowest M values were taken as having the most stable expression [43].

The relative expression level of each candidate gene in a virus-infected sample (target) was analyzed over the virus-free sample (calibrator) using the *geNorm *software [43]. Briefly, the sample with the lowest C_q _value was assigned the value 1, and raw C_q _values were calculated using the delta-C_q _formula *Q *= *E^ΔCq^*, where *E *is the primer efficiency and ΔC_q _is the sample with the highest expression (minimum C_q _value) from the data set minus C_q _value of the sample in question. The raw C_q _value (i.e. non-normalized) for each candidate gene in each sample was divided by the normalization factor (NF). Subsequently, the normalized value for each candidate gene in the target was divided by the normalized value for the corresponding gene in the calibrator to generate relative expression of flavonoid biosynthetic pathway genes in virus-infected leaves. The relative expression value for each gene represents mean of five biological replicates, with each replicate, in turn, representing a mean of three technical replicates. Each technical replicate, in turn, is a mean of duplicate values.

### Extraction and HPLC analysis of anthocyanins and flavonols

Anthocyanins and flavonols were extracted and subsequently analyzed by reverse-phase high performance liquid chromatography (HPLC) as described by Downey and Rochfort with slight modifications [79]. Frozen leaf samples were ground into fine powder in a mortar using liquid N_2 _and 100 mg powder per sample was added separately to 1 ml of 50% methanol. The samples were sonicated for 20 min, clarified by centrifugation at 13,000 g for 10 min and the supernatant filtered through a 0.22 uM Nylon Costar Spin-X Centrifuge Tube Filters (Corning Incorporated, Corning, NY, USA). The filtrate was directly transferred to 1.5 ml brown vials and analyzed for anthocyanins and flavonols. The HPLC system consisted of an Agilent 1100 series with a quaternary pump, coupled with diode array and multiple wavelength detectors (Palo Alto, CA). Column temperature was maintained at 40°C and separation occurred under the following conditions and gradients: solvent A, water/formic acid (90:10); solvent B, methanol/formic acid (90:10); flow rate at 1.0 ml/min; column: C-18 SS Wakosil (150 mm×4.6 mm, 3 m packing; SGE, Ringwood, Australia) protected by an SGE C-18 guard column of the same packing material; gradient program: 0 min 6% B, 10 min 12% B, 15 min 18% B, 20 min 24% B, 30 min 30% B and 45 min 45% B. Anthocyanins and flavonols were monitored by photodiode array detection (DAD) with the detection wavelength set at 520 nm and 353 nm, respectively. Malvidin-3-glucoside (Extrasynthese Co., Genay, France), cyanidin-3-glucoside (Extrasynthese Co., Genay, France) and quercetin-3-glucuronide (Sigma-Aldrich, St Louis, MO, USA) were quantified with their respective standard curves over three orders of magnitudes, with linear correlation coefficients greater than 0.999. Myricetin-3-glucoside, quercetin-3-glucoside and peonidin-3-glucoside-*p*-coumarate were putatively identified according to spectra and retention time. Five biological replicates (virus-infected and virus-free leaves collected from five individual grapevines for each category) were used for these analyses and measurements for each sample were carried out in duplicate.

### Estimation of proanthocyanidins

Proanthocyanidins (PAs) were extracted from leaves (collected from five virus-infected and five virus-free grapevines) as described in Harbertson *et al*. with some modifications and estimated as total tannins [80]. Briefly, 100 mg of frozen leaf tissue was extracted in 5 ml of 70% aqueous acetone (v/v) for 12 hours and filtered using Whatman No. 1 filter paper. Aqueous extract containing PAs was collected after removal of acetone using a rotary evaporator (Buchi Syncore, Buchi Switzerland) at 40°C and 525 mm Hg pressure. PAs were precipitated and resuspended in an alkaline detergent buffer and reacted subsequently with ferric chloride. The resulting reaction was monitored after 10 min at 510 nm using a Beckman DU 640 spectrophotometer (Beckman Instruments, St. Louis USA). A standard curve was developed using known amounts of (+)-catechin (a PA sub-unit) reacted with ferric chloride in an alkaline detergent buffer to interpret PA values. Concentration of PAs in leaf samples were reported in catechin equivalents (C.E.).

### Statistical analysis

Differences in total chlorophylls and carotenoids, total anthocyanins, total flavonols, total proanthocyanidns and relative gene expression values between virus-infected and virus-free leaves were analyzed by one-way ANOVA, using the SigmaPlot 11 software. The confidence level of all analyses was set at 95% and values with *p *≤ 0.05 were considered significant.

## Authors' contributions

RAN conceived and coordinated the study. RAN and LRG designed the research. LRG collected samples, performed experiments related to gene expression by RT-qPCR and extraction of pigments, and analyzed the data. LFC and LRG performed HPLC analysis, and LFC and JFH analyzed the HPLC results. RAN and LRG wrote the manuscript with contributions from LFC and JFH. All authors read and approved the final manuscript.

## Supplementary Material

Additional file 1 **Figure S1. Detection of GLRaV-3 in green veins and reddish-purple inter-veinal areas of virus-infected grapevine leaves by single tube RT-PCR**. L and V represent reddish-purple inter-veinal areas and green veins, respectively, and 1408, 1508, 1409, 1509, 3109 are code numbers for virus-infected grapevines. Lanes N and P represent negative and positive controls, respectively, for GLRaV-3. Lane M represents DNA molecular weight markers used to estimate the size of virus-specific DNA fragment amplified by RT-PCR. The 546 nucleotide DNA band amplified in test samples (indicated by arrow on the right) represents a portion of the 70-kDa heat-shock protein homolog of GLRaV-3 [32,75]. Click here for file

Additional file 2 **Figure S2. Melting curve analysis of gene-specific amplicons**. The blue colored horizontal line indicates base line generated with no template control and the red colored curve indicates dissociation curve for each gene. See legends for Figure 1 and 3 for names of genes. Click here for file

Additional file 3 **Figure S3. Box plot representation of raw C_q _values obtained from amplification curves for the flavonoid biosynthetic pathway genes in GLRaV-3-infected and virus-free leaves**. Lower and upper boundaries of each box indicate the 25^th ^and the 75^th ^percentile, respectively. Ranges are represented as bars (whiskers) below and above the box and indicate the 10^th ^and 90^th ^percentiles, respectively. The horizontal line in each box represents mean and outliers by (·). Suffix -D and -H for each gene denotes virus-infected and virus-free samples, respectively. See legend for Figure 1 for names of genes. Click here for file
