# Supplementary material for: Modulation of flavonoid biosynthetic pathway genes and anthocyanins due to virus infection in grapevine (Vitis vinifera L.) leaves
Source: BMC Plant Biol. 2010 Aug 23;10:187. doi: 10.1186/1471-2229-10-187 (PMC2956537; doi:10.1186/1471-2229-10-187)
Supplement: Supplementary file 2 [file 1471-2229-10-187-S2.DOC]

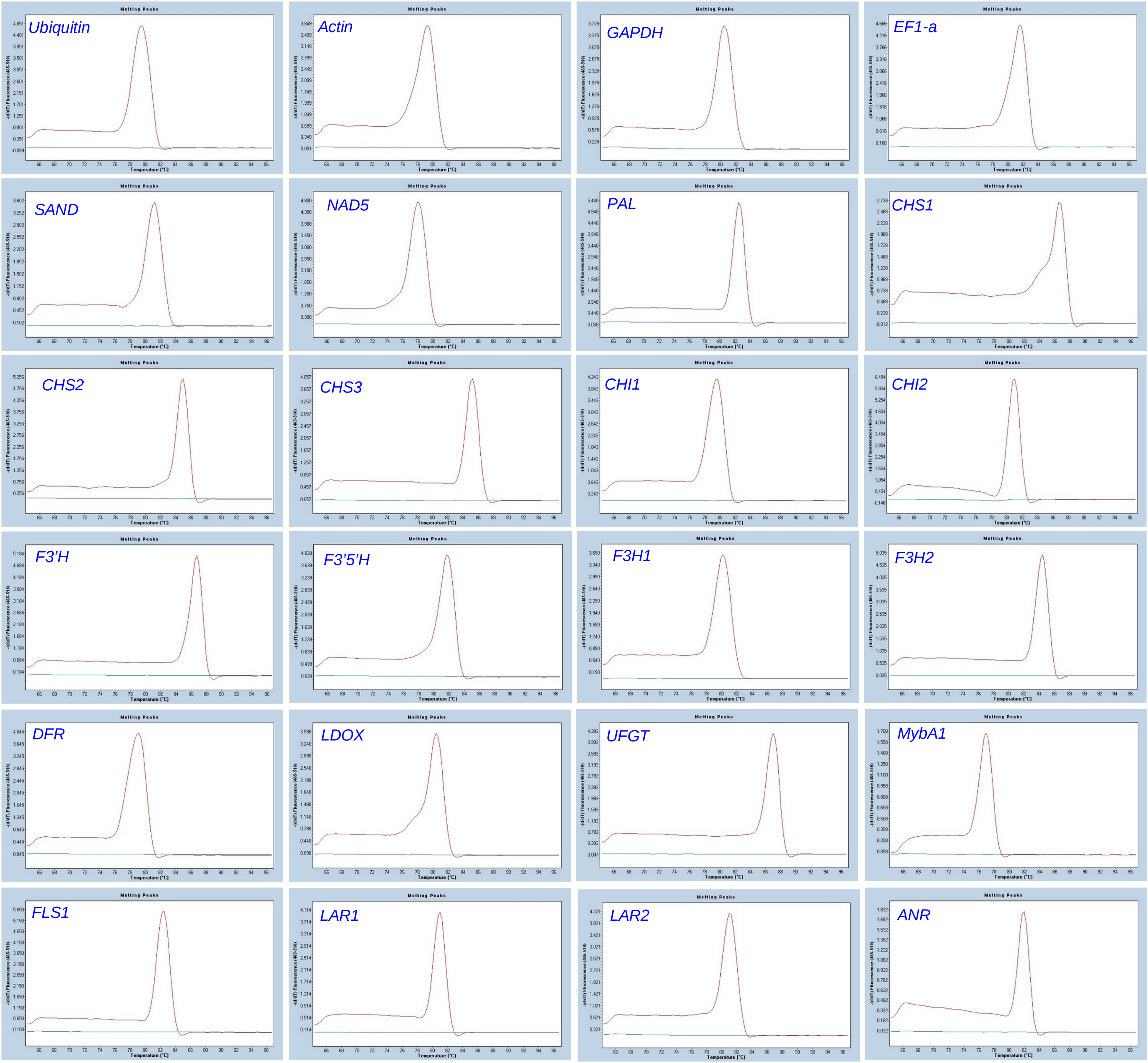
Melting Peaks
Temperature (°C)
Ubiquitin
Actin
GAPDH
EF1-a
SAND
NAD5
PAL
CHS1
CHS2
CHS3
CHI1
CHI2
F3'H
F3'5'H
F3H1
F3H2
DFR
LDOX
UFGT
MybA1
FLS1
LAR1
LAR2
ANR
