# Supplementary material for: Modulation of flavonoid biosynthetic pathway genes and anthocyanins due to virus infection in grapevine (Vitis vinifera L.) leaves
Source: BMC Plant Biol. 2010 Aug 23;10:187. doi: 10.1186/1471-2229-10-187 (PMC2956537; doi:10.1186/1471-2229-10-187)
Supplement: Supplementary file 3 [file 1471-2229-10-187-S3.DOC]

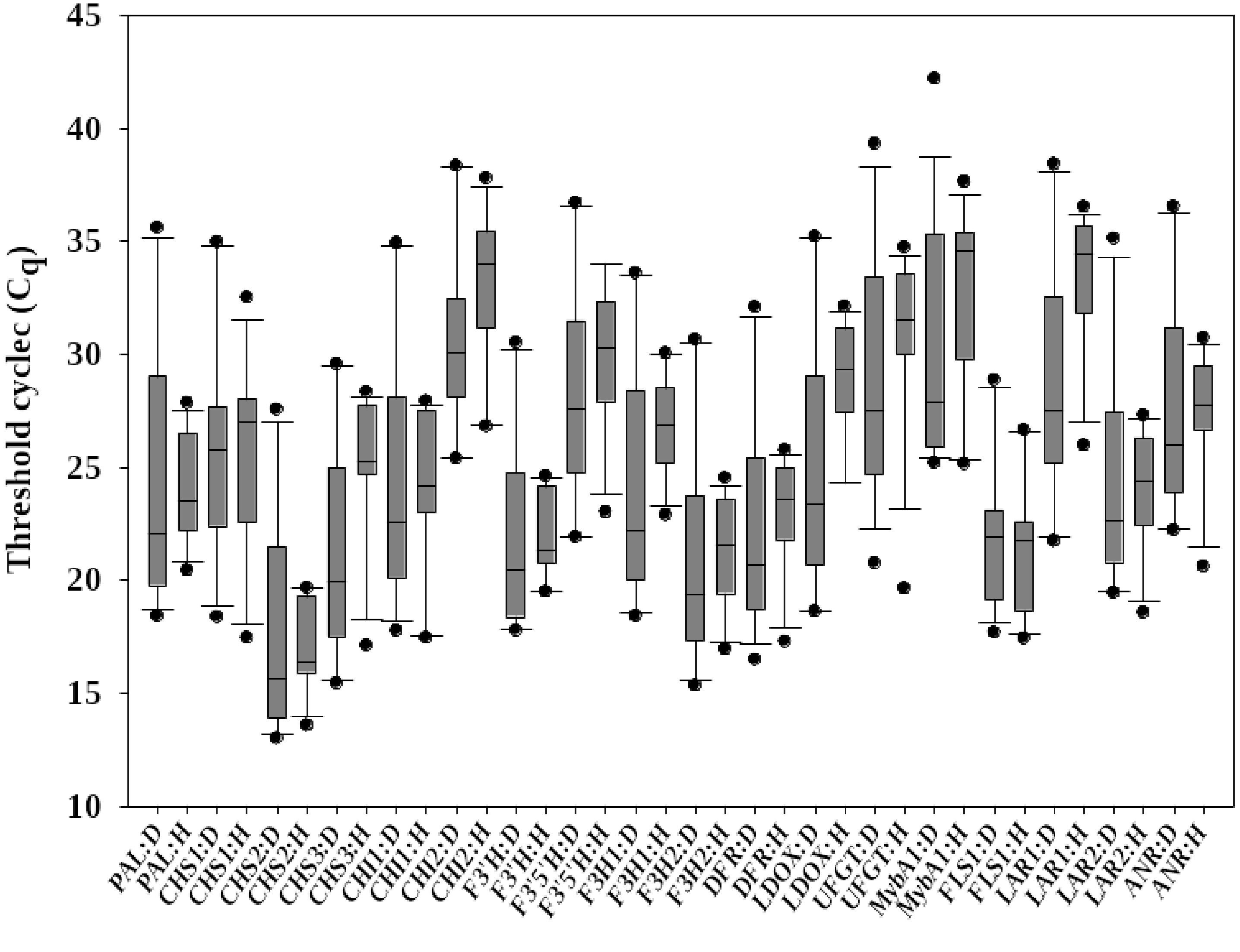

Threshold cyclec (C_q)
45
40
35
30
25
20
15
10
PAL:D
PAL:H
CHS1:D
CHS1:H
CHS2:D
CHS2:H
CHS3:D
CHS3:H
CHI1:D
CHI1:H
CHI2:D
CHI2:H
F3'H:D
F3'H:H
F3'5'H:D
F3'5'H:H
F3H1:D
F3H1:H
F3H2:D
F3H2:H
DFR:D
DFR:H
LDOX:D
LDOX:H
UFGT:D
UFGT:H
MybA1:D
MybA1:H
FLS1:D
FLS1:H
LAR1:D
LAR1:H
LAR2:D
LAR2:H
ANR:D
ANR:H
